# Supplementary material for: AntiBP3: A Method for Predicting Antibacterial Peptides against Gram-Positive/Negative/Variable Bacteria
Source: Antibiotics (Basel). 2024 Feb 8;13(2):168. doi: 10.3390/antibiotics13020168 (PMC10885866; doi:10.3390/antibiotics13020168)
Supplement: Supplementary file 1 [file antibiotics-13-00168-s001.zip › Figure S1.pdf]

Figure S1: AUC-ROC comparison of different ML models on compositional-based AAC features for GP, GN and GV ABPs.

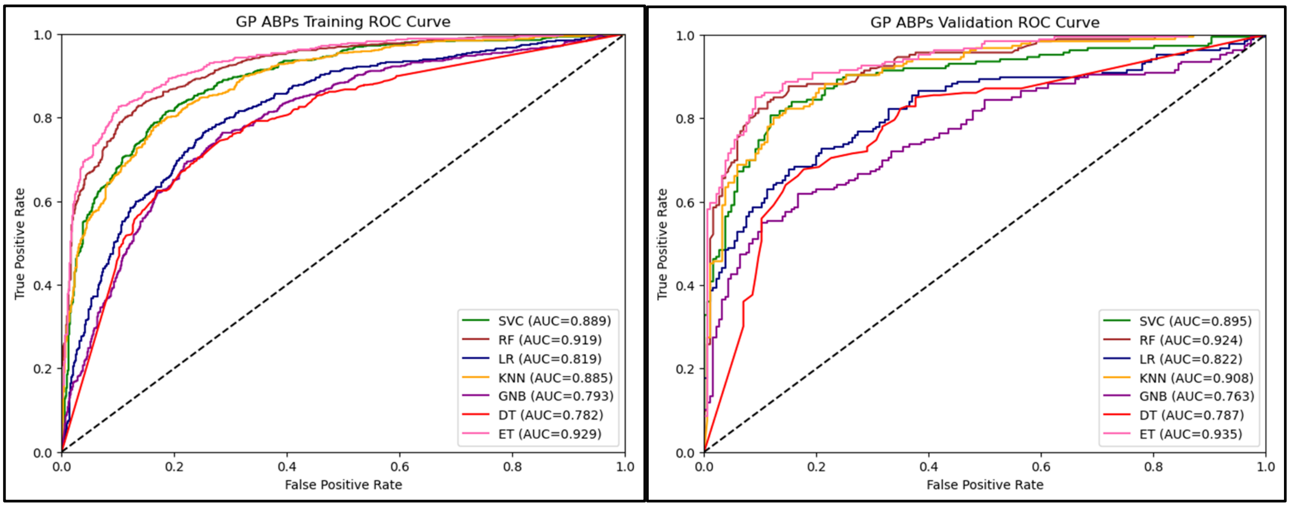

Figure S1.1: The performance of ML models on training and testing data of GP ABPs on AAC feature

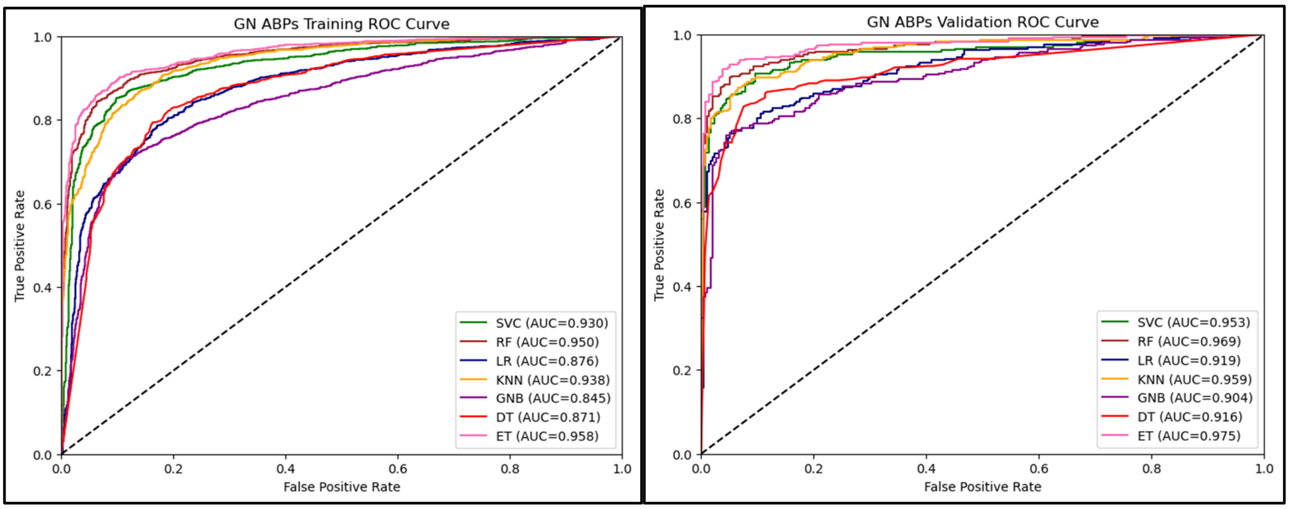

Figure S1.2: The performance of ML models on training and testing data of GN ABPs on AAC feature

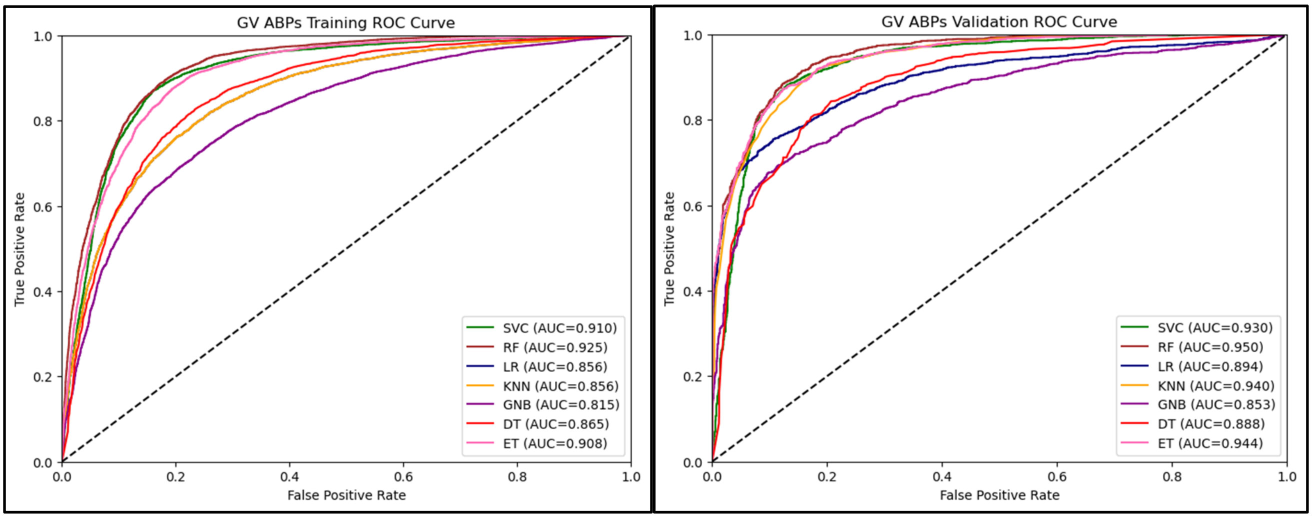

Figure S1.3: The performance of ML models on training and testing data of GV ABPs on AAC feature
