# Supplementary material for: AntiBP3: A Method for Predicting Antibacterial Peptides against Gram-Positive/Negative/Variable Bacteria
Source: Antibiotics (Basel). 2024 Feb 8;13(2):168. doi: 10.3390/antibiotics13020168 (PMC10885866; doi:10.3390/antibiotics13020168)
Supplement: Supplementary file 1 [file antibiotics-13-00168-s001.zip › Figure S2.pdf]

Figure S2: The AUC-ROC comparison of different ML models on binary-profile AAB features for NC-terminal for GP, GN and GV ABPs.

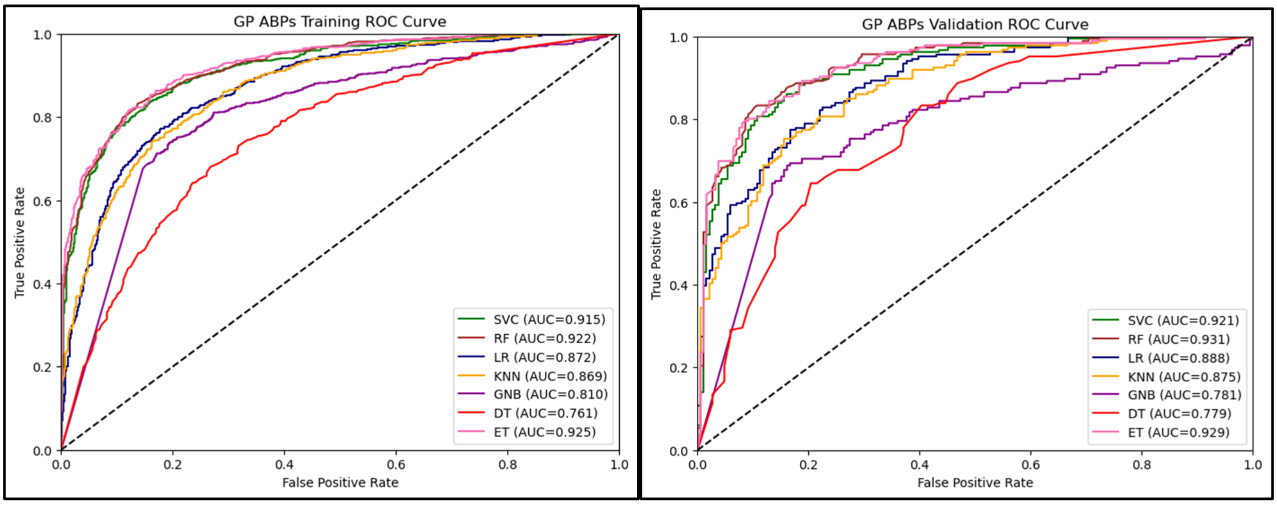

Figure S2.1: The performance of ML models on training and testing data of GP ABPs on AAB (NC-terminal)

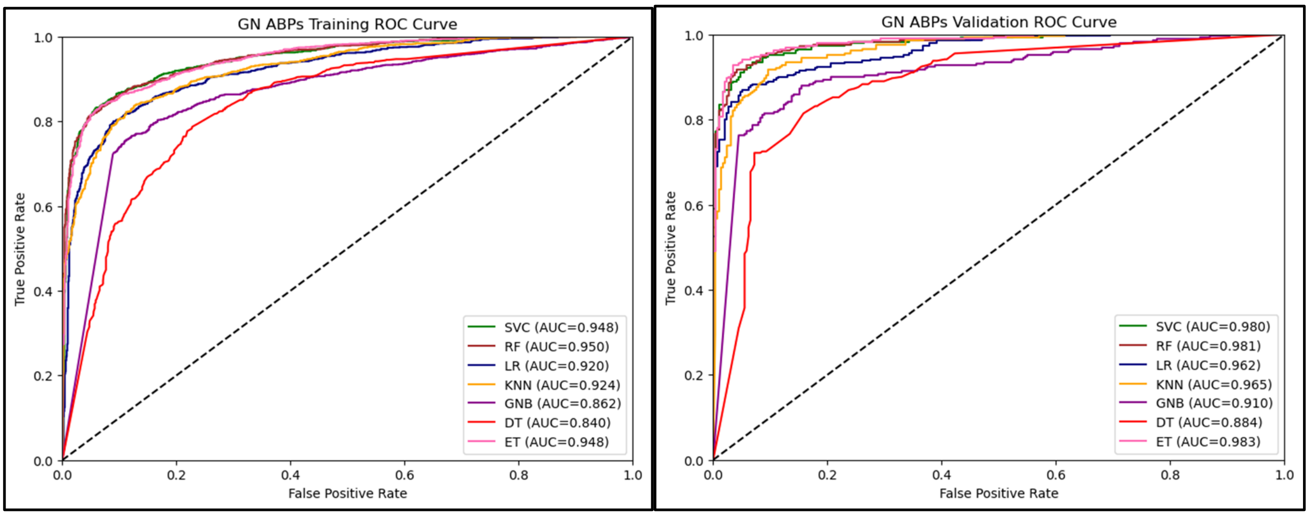

Figure S2.2: Performance of ML models on training and testing data of GN ABPs on AAB (NC-terminal)

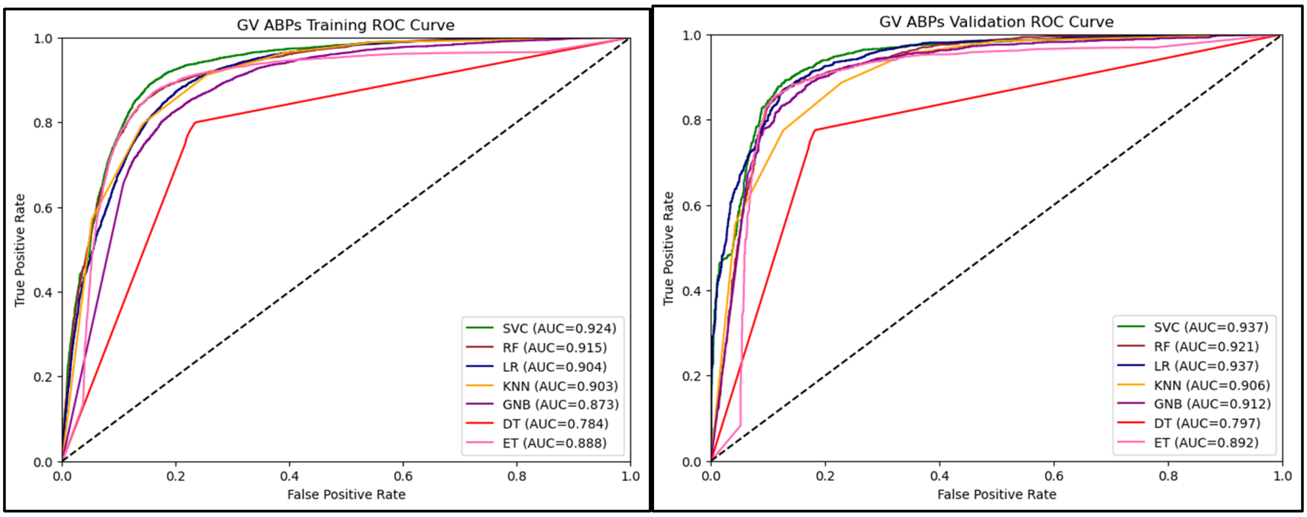

Figure S2.3: Performance of ML models on training and testing data of GV ABPs on AAB (NC-terminal)
