# Supplementary material for: AntiBP3: A Method for Predicting Antibacterial Peptides against Gram-Positive/Negative/Variable Bacteria
Source: Antibiotics (Basel). 2024 Feb 8;13(2):168. doi: 10.3390/antibiotics13020168 (PMC10885866; doi:10.3390/antibiotics13020168)

Figure S3: Confusion matrix of best ML-model (AAB-based binary feature for NC-terminal) on the validation set of GP, GN and GV ABPs independent

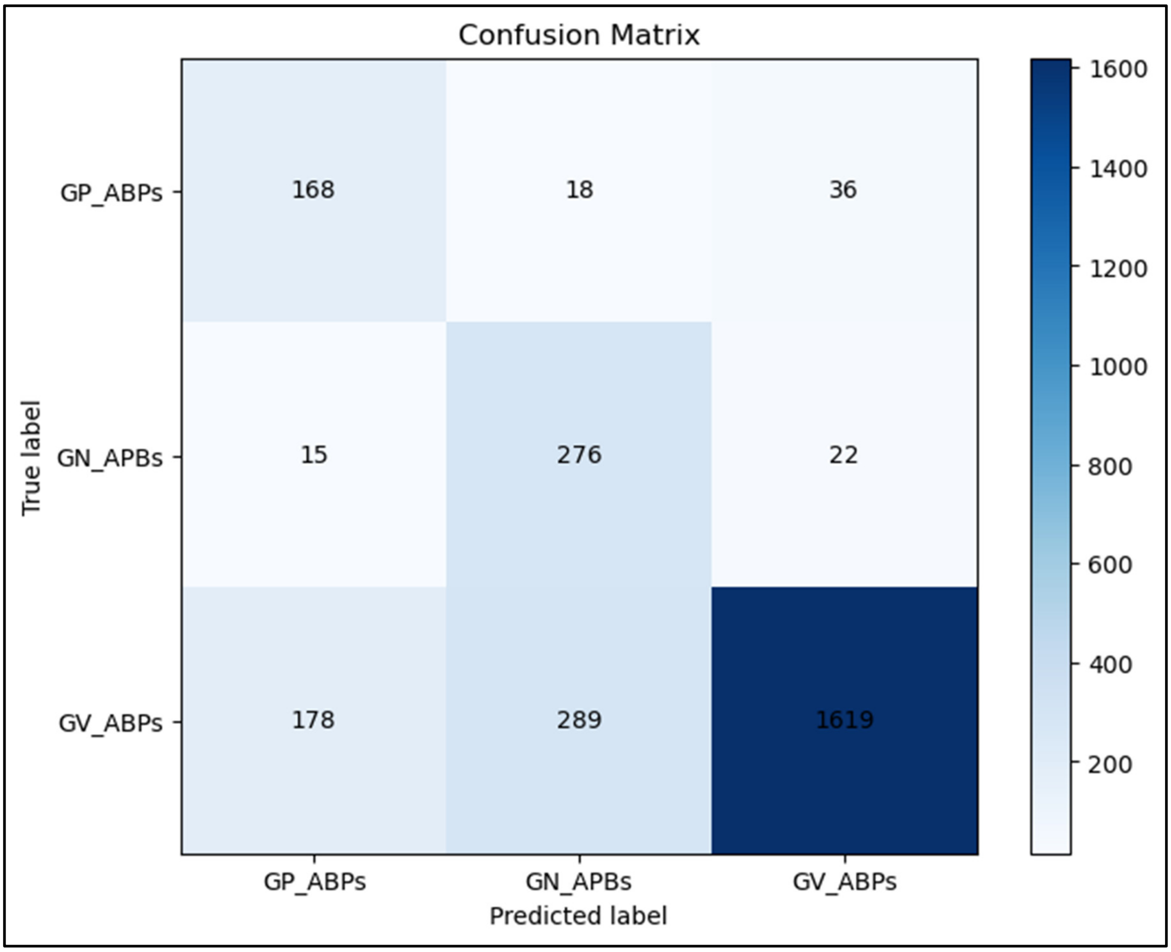

Supplement: Supplementary file 1 [file antibiotics-13-00168-s001.zip › Figure S3.pdf]
