# Supplementary material for: AntiBP3: A Method for Predicting Antibacterial Peptides against Gram-Positive/Negative/Variable Bacteria
Source: Antibiotics (Basel). 2024 Feb 8;13(2):168. doi: 10.3390/antibiotics13020168 (PMC10885866; doi:10.3390/antibiotics13020168)
Supplement: Supplementary file 1 [file antibiotics-13-00168-s001.zip › Table S10.pdf]

Table S10: Performance of hybrid model (BLAST+ML) on the validation set of gram-variable ABPs independent at different e-value

E-value	Correct hits (BLAST+ML)	Sn	Sp	Acc	AUC	AUPRC	MCC
**1.00E-20**	87.26%	86.5	88.6	87.6	0.93	0.92	0.75
**1.00E-10**	87.37%	87.2	88.3	87.7	0.92	0.87	0.75
**1.00E-06**	87.59%	86.8	88.9	87.8	0.92	0.86	0.76
**0.0001**	87.62%	86.8	88.8	87.8	0.92	0.86	0.76
**0.001**	87.62%	86.6	88.8	87.7	0.92	0.87	0.75
**0.01**	87.40%	86.1	88.9	87.5	0.92	0.87	0.75
**0.1**	87.34%	85.9	88.8	87.3	0.92	0.88	0.75
**1**	86.84%	85.1	88.3	86.7	0.92	0.88	0.73
**10**	83.81%	81.4	85.0	83.2	0.91	0.88	0.66
**50**	82.39%	79.2	84.1	81.7	0.91	0.88	0.63
**100**	82.03%	78.9	83.8	81.3	0.91	0.88	0.63
**200**	81.91%	78.8	83.6	81.2	0.91	0.88	0.63
**1000**	81.80%	78.6	83.6	81.1	0.91	0.88	0.62
**Only ML**	87.01%	83.9	90.1	87.0	0.94	0.93	0.74

Sn: Sensitivity, **Sp:** Specificity, **Acc:** Accuracy, **MCC:** Matthews Correlation Coefficient, **AUC:** Area Under the Receiver Operating Characteristic curve, **AUPRC:** Area Under the Precision-Recall Curve
