# Supplementary material for: AntiBP3: A Method for Predicting Antibacterial Peptides against Gram-Positive/Negative/Variable Bacteria
Source: Antibiotics (Basel). 2024 Feb 8;13(2):168. doi: 10.3390/antibiotics13020168 (PMC10885866; doi:10.3390/antibiotics13020168)
Supplement: Supplementary file 1 [file antibiotics-13-00168-s001.zip › Table S11.pdf]

Table S11: Performance of hybrid model (MOTIF+ML) on the validation set of gram-positive ABPs independent set at different frequencies

Frequency of a motif	Correct hits (MOTIF+ML)	Sn	Sp	Acc	AUC	AUPRC	MCC
**fp10_motif**	84.68%	83.3	86.0	84.7	0.91	0.88	0.69
**fp20_motif**	85.22%	81.7	88.7	85.2	0.92	0.90	0.71
**fp30_motif**	85.22%	81.2	89.3	85.2	0.92	0.90	0.71
**Only ML**	**85.48%**	**80.7**	**90.3**	**85.5**	**0.93**	**0.92**	**0.71**

Sn: Sensitivity, **Sp:** Specificity, **Acc:** Accuracy, **MCC:** Matthews Correlation Coefficient, **AUC:** Area Under the Receiver Operating Characteristic curve, **AUPRC:** Area Under the Precision-Recall Curve
