# Supplementary material for: AntiBP3: A Method for Predicting Antibacterial Peptides against Gram-Positive/Negative/Variable Bacteria
Source: Antibiotics (Basel). 2024 Feb 8;13(2):168. doi: 10.3390/antibiotics13020168 (PMC10885866; doi:10.3390/antibiotics13020168)
Supplement: Supplementary file 1 [file antibiotics-13-00168-s001.zip › Table S12.pdf]

Table S12: Performance of hybrid model (MOTIF+ML) on the validation set of gram-negative ABPs independent set at different frequencies

Frequency of a motif	Correct hits (MOTIF+ML)	Sn	Sp	Acc	AUC	AUPRC	MCC
**fp10_motif**	90.21%	93.5	86.9	90.2	0.96	0.95	0.81
**fp20_motif**	92.27%	92.8	91.8	92.3	0.97	0.97	0.85
**fp30_motif**	93.30%	92.8	93.8	93.3	0.97	0.97	0.87
**Only ML**	**93.64%**	**92.4**	**94.9**	**93.6**	**0.98**	**0.98**	**0.86**

# Sn: Sensitivity, **Sp:** Specificity, **Acc:** Accuracy, **MCC:** Matthews Correlation Coefficient, **AUC:** Area Under the Receiver Operating Characteristic curve, **AUPRC:** Area Under the Precision-Recall Curve
