# Supplementary material for: AntiBP3: A Method for Predicting Antibacterial Peptides against Gram-Positive/Negative/Variable Bacteria
Source: Antibiotics (Basel). 2024 Feb 8;13(2):168. doi: 10.3390/antibiotics13020168 (PMC10885866; doi:10.3390/antibiotics13020168)
Supplement: Supplementary file 1 [file antibiotics-13-00168-s001.zip › Table S13.pdf]

**Table S13: Performance of hybrid model (MOTIF+ML) on the validation set of gram-variable ABPs independent set at different frequencies**

| Frequency of a motif | Correct hits (MOTIF+ML) | Sn | Sp | Acc | AUC | AUPRC | MCC |
|---|---|---|---|---|---|---|---|
| **fp10_motif** | 86.03% | 86.7 | 85.4 | 86.0 | 0.93 | 0.92 | 0.72 |
| **fp20_motif** | 87.01% | 85.8 | 88.3 | 87.0 | 0.94 | 0.94 | 0.74 |
| **fp30_motif** | 87.28% | 85.5 | 89.1 | 87.3 | 0.94 | 0.93 | 0.75 |
| **Only ML** | **87.01%** | **83.9** | **90.1** | **87.0** | **0.94** | **0.93** | **0.74** |

# **Sn:** Sensitivity, **Sp:** Specificity, **Acc:** Accuracy, **MCC:** Matthews Correlation Coefficient, **AUC:** Area Under the Receiver Operating Characteristic curve, **AUPRC:** Area Under the Precision-Recall Curve
