# Supplementary material for: AntiBP3: A Method for Predicting Antibacterial Peptides against Gram-Positive/Negative/Variable Bacteria
Source: Antibiotics (Basel). 2024 Feb 8;13(2):168. doi: 10.3390/antibiotics13020168 (PMC10885866; doi:10.3390/antibiotics13020168)
Supplement: Supplementary file 1 [file antibiotics-13-00168-s001.zip › Table S14.pdf]

Table S14: Performance of DL-models on validation dataset developed using composition-based features for gram-positive ABPs

DL Model	ANN			CNN			RNN			LSTM		
**Feature Type**	**AUC**	**AUPRC**	**MCC**	**AUC**	**AUPRC**	**MCC**	**AUC**	**AUPRC**	**MCC**	**AUC**	**AUPRC**	**MCC**
**AAC**	0.84	0.86	0.53	0.87	0.88	0.57	0.90	0.91	0.69	0.89	0.90	0.64
**DPC**	0.87	0.88	0.62	0.88	0.88	0.60	0.88	0.89	0.64	0.90	0.88	0.62
**ATC**	0.64	0.65	0.29	0.65	0.66	0.09	0.61	0.65	0.14	0.59	0.66	0.16
**BTC**	0.67	0.60	0.04	0.66	0.60	0.04	0.68	0.60	0.17	0.37	0.42	-0.06
**CTC**	0.85	0.86	0.59	0.86	0.86	0.61	0.85	0.86	0.59	0.85	0.86	0.58
**PCP**	**0.90**	0.91	0.66	0.85	0.88	0.54	**0.92**	0.92	0.69	0.79	0.78	0.44
**RRI**	0.83	0.85	0.53	0.85	0.84	0.54	0.86	0.84	0.53	0.82	0.83	0.50
**PRI**	0.71	0.74	0.33	0.82	0.83	0.46	0.82	0.80	0.48	0.73	0.75	0.36
**DDR**	0.81	0.79	0.48	0.82	0.80	0.40	0.84	0.83	0.55	0.71	0.71	0.32
**SEP**	0.72	0.72	0.41	0.72	0.72	0.25	0.72	0.72	0.23	0.72	0.72	0.22
**SER**	0.87	0.88	0.58	0.88	0.88	0.58	0.90	0.90	0.72	0.85	0.86	0.50
**SPC**	0.81	0.85	0.47	0.84	0.85	0.50	0.87	0.89	0.62	0.78	0.79	0.43
**PAAC**	0.86	0.84	0.60	0.89	0.90	0.58	0.87	0.88	0.63	0.89	0.84	0.63
**APAAC**	0.87	0.86	0.58	0.88	0.87	0.59	0.89	0.90	0.61	0.88	0.89	0.66
**QSO**	0.87	0.87	0.62	**0.91**	0.92	0.67	0.88	0.89	0.58	**0.90**	0.90	0.66

AUC: Area Under the Receiver Operating Characteristic curve, **AUPRC:**Area Under the Precision-Recall Curve, **MCC:** Matthews Correlation Coefficient, **AAC:** Amino acid

composition, **APAAC:** Amphiphilic pseudo amino acid composition, **DDR:** Distance distribution of residue, **DPC:** Di-peptide composition, **QSO:** Quasi-sequence order, **PCP:** Physico-chemical properties composition, **PAAC:** Pseudo amino acid composition, **RRI:** Residue repeat Information, **SPC:** Shannon entropy of physicochemical properties, **ATC:** Atomic composition , **BTC:** Bond type composition, **CTC:** Conjoint triad descriptors, **PRI:** Property repeats index, **SEP:** Shannon entropy of a protein, **SER:** Shannon entropy of a residue, **ANN:** Artificial neural network, **CNN:** Convolution neural network, **RNN:** Recurrent neural network, **LSTM:** Long short-term memory
