# Supplementary material for: AntiBP3: A Method for Predicting Antibacterial Peptides against Gram-Positive/Negative/Variable Bacteria
Source: Antibiotics (Basel). 2024 Feb 8;13(2):168. doi: 10.3390/antibiotics13020168 (PMC10885866; doi:10.3390/antibiotics13020168)
Supplement: Supplementary file 1 [file antibiotics-13-00168-s001.zip › Table S15.pdf]

Table S15: Performance of DL-models on validation dataset developed using four types of Binary profile-based features of N, C, NC-terminal of gram-positive ABPs

Feature type	Terminal	ANN			CNN			RNN			LSTM		
		AUC	AUPRC	MCC	AUC	AUPRC	MCC	AUC	AUPRC	MCC	AUC	AUPRC	MCC
AAB	N	0.88	0.88	0.60	0.88	0.88	0.58	0.87	0.88	0.63	0.85	0.86	0.55
	C	0.83	0.84	0.48	0.81	0.81	0.46	0.83	0.83	0.50	0.80	0.79	0.47
	NC	0.89	0.89	0.62	0.89	0.90	0.61	0.88	0.89	0.64	0.85	0.86	0.55
DPB	N	0.87	0.89	0.55	0.89	0.89	0.64	0.87	0.88	0.62	0.88	0.87	0.60
	C	0.84	0.84	0.51	0.84	0.84	0.51	0.83	0.82	0.49	0.83	0.84	0.47
	NC	**0.90**	0.90	0.64	0.88	0.89	0.62	**0.90**	0.89	0.65	0.87	0.88	0.59
AIB	N	0.88	0.86	0.61	0.88	0.87	0.63	0.88	0.88	0.61	0.88	0.89	0.61
	C	0.82	0.81	0.49	0.82	0.80	0.48	0.84	0.83	0.55	0.80	0.80	0.46
	NC	0.90	0.88	0.68	0.89	0.88	0.66	**0.90**	0.89	0.64	**0.89**	0.89	0.62
PCB	N	0.89	0.90	0.65	**0.90**	0.89	0.65	0.89	0.90	0.65	0.87	0.87	0.60
	C	0.84	0.83	0.54	0.81	0.80	0.47	0.81	0.79	0.44	0.81	0.80	0.48
	NC	0.90	0.89	0.67	0.88	0.86	0.64	0.89	0.89	0.64	0.87	0.87	0.57

MCC: Matthews Correlation Coefficient, **AUC:** Area Under the Receiver Operating Characteristic curve, **AUPRC:** Area Under the Precision-Recall Curve, **AAB:** Amino acid-based binary profile, **DPB:** Dipeptide-based binary profile, **PCB:** Physico-chemical properties based binary profile, **AIB:** Amino-acid indices based binary profile, **ANN:** Artificial neural network, **CNN:** Convolution neural network, **RNN:** Recurrent neural network, **LSTM:** Long short-term memory
