# Supplementary material for: AntiBP3: A Method for Predicting Antibacterial Peptides against Gram-Positive/Negative/Variable Bacteria
Source: Antibiotics (Basel). 2024 Feb 8;13(2):168. doi: 10.3390/antibiotics13020168 (PMC10885866; doi:10.3390/antibiotics13020168)
Supplement: Supplementary file 1 [file antibiotics-13-00168-s001.zip › Table S16.pdf]

Table S16: Performance of DL-models on validation dataset developed using FastText based features for GP ABPs

Feature (n-gram size)	ANN			CNN			RNN			LSTM		
	AUC	AUPRC	MCC	AUC	AUPRC	MCC	AUC	AUPRC	MCC	AUC	AUPRC	MCC
**1g**	0.85	0.81	0.56	0.85	0.85	0.52	**0.87**	0.87	0.56	0.79	0.79	0.42
**2g**	**0.88**	0.85	0.64	0.86	0.85	0.56	0.86	0.84	0.53	**0.86**	0.87	0.59
**3g**	0.86	0.87	0.56	0.82	0.84	0.50	0.84	0.86	0.56	0.83	0.84	0.52
**2g & 3g combined**	0.87	0.88	0.59	**0.88**	0.86	0.63	0.85	0.86	0.54	0.83	0.82	0.55

AUC: Area Under the Receiver Operating Characteristic curve, **AUPRC:** Area Under the Precision-Recall Curve**,** **MCC:** Matthews Correlation Coefficient, **ANN:** Artificial neural network, **CNN:** Convolution neural network, **RNN:** Recurrent neural network, **LSTM:** Long short-term memory
