# Supplementary material for: AntiBP3: A Method for Predicting Antibacterial Peptides against Gram-Positive/Negative/Variable Bacteria
Source: Antibiotics (Basel). 2024 Feb 8;13(2):168. doi: 10.3390/antibiotics13020168 (PMC10885866; doi:10.3390/antibiotics13020168)
Supplement: Supplementary file 1 [file antibiotics-13-00168-s001.zip › Table S2.pdf]

Table S2: Performance of Extra-tree classifier on training and validation dataset developed using 17 types of composition-based features for gram-negative ABPs

Feature Type	Training set						Validation set					
	Sn	Sp	Acc	AUC	AUPRC	MCC	Sn	Sp	Acc	AUC	AUPRC	MCC
**AAC**	90.0	89.6	89.8	0.96	0.96	0.80	93.1	93.1	93.1	0.98	0.98	0.86
**DPC**	88.2	88.3	88.3	0.95	0.96	0.77	94.5	91.4	93.0	0.98	0.98	0.86
**ATC**	84.1	84.5	84.3	0.91	0.92	0.69	88.7	89.7	89.2	0.95	0.96	0.78
**BTC**	71.4	71.1	71.3	0.79	0.79	0.43	76.6	74.6	75.6	0.84	0.86	0.51
**CTC**	87.5	88.0	87.7	0.94	0.94	0.75	92.1	90.0	91.1	0.97	0.98	0.82
**PCP**	88.3	87.9	88.1	0.95	0.95	0.76	91.1	92.8	91.9	0.97	0.98	0.84
**AAI**	88.5	88.0	88.2	0.95	0.95	0.77	92.4	93.1	92.8	0.97	0.98	0.86
**RRI**	87.5	87.2	87.4	0.94	0.95	0.75	89.0	93.1	91.1	0.97	0.97	0.82
**PRI**	86.3	85.8	86.1	0.94	0.94	0.72	90.0	89.7	89.9	0.97	0.97	0.80
**DDR**	86.7	86.7	86.7	0.94	0.95	0.73	88.3	91.4	89.9	0.97	0.97	0.80
**SEP**	70.0	66.7	68.3	0.72	0.68	0.37	69.8	75.6	73.7	0.80	0.63	0.44
**SER**	89.2	88.9	89.1	0.96	0.96	0.78	91.1	94.9	93.0	0.97	0.98	0.86
**SPC**	87.6	87.9	87.8	0.94	0.94	0.76	90.7	90.0	90.4	0.96	0.97	0.81
**PAAC**	90.1	89.8	90.0	0.96	0.96	0.80	92.8	94.2	93.5	0.97	0.98	0.87
**APAAC**	89.4	89.6	89.5	0.96	0.96	0.79	92.1	94.5	93.3	0.98	0.98	0.87

**QSO**	89.2	89.4	89.3	0.96	0.96	0.79	90.7	93.5	92.1	0.97	0.98	0.84
**SOC**	69.4	69.3	69.4	0.76	0.76	0.39	72.2	70.5	71.3	0.79	0.80	0.43

Sn: Sensitivity, **Sp:** Specificity, **Acc:** Accuracy, **MCC:** Matthews Correlation Coefficient, **AUC:** Area Under the Receiver Operating Characteristic curve, **AUPRC:**Area Under the Precision-Recall Curve, **AAC:** Amino acid composition, **APAAC:** Amphiphilic pseudo amino acid composition, **DDR:** Distance distribution of residue, **DPC:** Di-peptide composition, **QSO:** Quasi-sequence order, **PCP:** Physico-chemical properties composition, **PAAC:** Pseudo amino acid composition, **RRI:** Residue repeat Information, **SPC:** Shannon entropy of physicochemical properties, **ATC:** Atomic composition , **BTC:** Bond type composition, **CTC:** Conjoint triad descriptors, **AAI:** Amino Acid index, **PRI:** Property repeats index, **SEP:** Shannon entropy of a protein, **SER:** Shannon entropy of a residue, **SOC:** Sequence order coupling number
