# Supplementary material for: AntiBP3: A Method for Predicting Antibacterial Peptides against Gram-Positive/Negative/Variable Bacteria
Source: Antibiotics (Basel). 2024 Feb 8;13(2):168. doi: 10.3390/antibiotics13020168 (PMC10885866; doi:10.3390/antibiotics13020168)
Supplement: Supplementary file 1 [file antibiotics-13-00168-s001.zip › Table S3.pdf]

Table S3: Performance of Extra-tree classifier on training and validation dataset developed using 17 types of composition-based features for gram-variable ABPs

Feature Type	Training set						Validation set					
	Sn	Sp	Acc	AUC	AUPRC	MCC	Sn	Sp	Acc	AUC	AUPRC	MCC
AAC	85.1	85.6	85.3	0.93	0.91	0.71	84.7	89.7	87.2	0.95	0.95	0.74
DPC	85.2	84.9	85.1	0.92	0.90	0.70	83.0	87.3	85.1	0.92	0.90	0.70
ATC	79.0	79.0	79.0	0.86	0.85	0.58	80.7	81.4	81.1	0.89	0.89	0.62
BTC	68.4	69.1	68.8	0.76	0.74	0.38	66.7	70.6	68.6	0.76	0.73	0.37
CTC	83.9	83.8	83.9	0.90	0.89	0.68	83.2	87.3	85.3	0.91	0.90	0.71
PCP	83.6	83.5	83.5	0.91	0.90	0.67	84.3	87.4	85.8	0.93	0.91	0.72
AAI	84.1	83.7	83.9	0.91	0.89	0.68	85.1	87.3	86.2	0.92	0.90	0.73
RRI	84.0	84.2	84.1	0.91	0.90	0.68	82.6	86.5	84.6	0.92	0.90	0.69
PRI	83.1	83.2	83.2	0.90	0.89	0.66	81.9	85.4	83.6	0.91	0.89	0.67
DDR	84.3	84.4	84.3	0.91	0.90	0.69	84.1	87.9	86.0	0.92	0.90	0.72
SEP	67.6	67.1	67.4	0.74	0.73	0.35	72.5	72.7	72.6	0.79	0.78	0.45
SER	85.2	85.4	85.3	0.92	0.91	0.71	84.1	88.2	86.2	0.93	0.91	0.72
SPC	83.5	83.0	83.3	0.90	0.89	0.67	83.5	87.3	85.4	0.92	0.91	0.71
PAAC	85.4	85.1	85.2	0.92	0.91	0.71	85.3	88.3	86.8	0.93	0.91	0.74
APAAC	85.6	85.4	85.5	0.92	0.91	0.71	85.4	89.7	87.5	0.95	0.95	0.75

**QSO**	85.3	85.5	85.4	0.92	0.90	0.71	84.3	88.4	86.3	0.93	0.91	0.73
**SOC**	57.4	57.2	57.3	0.61	0.62	0.15	59.9	59.7	59.8	0.64	0.65	0.20

Sn: Sensitivity, **Sp:** Specificity, **Acc:** Accuracy, **MCC:** Matthews Correlation Coefficient, **AUC:** Area Under the Receiver Operating Characteristic curve, **AUPRC:**Area Under the Precision-Recall Curve, **AAC:** Amino acid composition, **APAAC:** Amphiphilic pseudo amino acid composition, **DDR:** Distance distribution of residue, **DPC:** Di-peptide composition, **QSO:** Quasi-sequence order, **PCP:** Physico-chemical properties composition, **PAAC:** Pseudo amino acid composition, **RRI:** Residue repeat Information, **SPC:** Shannon entropy of physicochemical properties, **ATC:** Atomic composition , **BTC:** Bond type composition, **CTC:** Conjoint triad descriptors, **AAI:** Amino Acid index, **PRI:** Property repeats index, **SEP:** Shannon entropy of a protein, **SER:** Shannon entropy of a residue, **SOC:** Sequence order coupling number
