# Supplementary material for: AntiBP3: A Method for Predicting Antibacterial Peptides against Gram-Positive/Negative/Variable Bacteria
Source: Antibiotics (Basel). 2024 Feb 8;13(2):168. doi: 10.3390/antibiotics13020168 (PMC10885866; doi:10.3390/antibiotics13020168)
Supplement: Supplementary file 1 [file antibiotics-13-00168-s001.zip › Table S4.pdf]

Table S4: Performance of ML-models on training and validation dataset developed using four types of Binary profile-based features of N, C, NC-terminal of gram-positive ABPs

Feature type	Terminal	Training set						Validation set					
		**Sn**	**Sp**	**Acc**	**AUC**	**AUPRC**	**MCC**	**Sn**	**Sp**	**Acc**	**AUC**	**AUPRC**	**MCC**
**AAB (RF)**	**N**	81.3	80.9	81.1	0.90	0.91	0.62	78.0	88.2	83.1	0.92	0.92	0.67
	**C**	79.2	79.6	79.4	0.87	0.89	0.59	75.3	79.6	77.4	0.86	0.86	0.55
	**NC**	84.1	84.1	84.1	0.92	0.92	0.68	80.7	90.3	85.5	**0.93**	0.92	0.71
**DPB (SVC)**	**N**	79.8	79.2	79.5	0.88	0.89	0.59	77.4	85.5	81.5	0.89	0.90	0.63
	**C**	77.0	76.9	77.0	0.85	0.88	0.54	68.8	83.3	76.1	0.85	0.85	0.53
	**NC**	82.3	81.9	82.1	0.90	0.91	0.64	79.0	88.2	83.6	**0.91**	0.90	0.68
**AIB (ET)**	**N**	79.8	79.0	79.4	0.89	0.90	0.59	78.0	86.6	82.3	0.91	0.91	0.65
	**C**	78.2	78.1	78.2	0.88	0.89	0.56	79.0	77.4	78.2	0.88	0.87	0.57
	**NC**	82.8	82.1	82.5	0.91	0.91	0.65	80.1	88.2	84.1	**0.93**	0.91	0.69
**PCB (RF)**	**N**	82.5	81.2	81.9	0.90	0.91	0.64	77.4	85.5	81.5	0.92	0.92	0.63
	**C**	79.8	80.9	80.4	0.88	0.89	0.61	75.8	80.7	78.2	0.86	0.85	0.57
	**NC**	82.9	81.5	82.2	0.91	0.91	0.64	82.3	87.6	85.0	**0.93**	0.92	0.70

Sn: Sensitivity, **Sp:** Specificity, **Acc:** Accuracy, **MCC:** Matthews Correlation Coefficient, **AUC:** Area Under the Receiver Operating Characteristic curve, **AUPRC:** Area Under the Precision-Recall Curve, **AAB:** Amino acid-based binary profile, **DPB:** Dipeptide-based binary profile, **PCB:** Physico-chemical properties based binary profile, **AIB:** Amino-acid indices based binary profile, **RF:** Random Forest classifier, **ET:** Extra-tree classifier, **SVC:** Support vector classifier
