# Supplementary material for: AntiBP3: A Method for Predicting Antibacterial Peptides against Gram-Positive/Negative/Variable Bacteria
Source: Antibiotics (Basel). 2024 Feb 8;13(2):168. doi: 10.3390/antibiotics13020168 (PMC10885866; doi:10.3390/antibiotics13020168)
Supplement: Supplementary file 1 [file antibiotics-13-00168-s001.zip › Table S5.pdf]

Table S5: Performance of ML-models on training and validation dataset developed using four types of Binary profile-based features of N, C, NC-terminal of gram-negative ABPs

Feature type	Terminal	Training set						Validation set					
		**Sn**	**Sp**	**Acc**	**AUC**	**AUPRC**	**MCC**	**Sn**	**Sp**	**Acc**	**AUC**	**AUPRC**	**MCC**
**AAB (ET)**	**N**	86.7	87.0	86.9	0.94	0.95	0.74	89.4	95.5	92.4	0.97	0.97	0.85
	**C**	83.8	84.2	84.0	0.92	0.93	0.68	89.7	89.4	89.5	0.95	0.96	0.79
	**NC**	86.8	88.0	87.4	0.95	0.95	0.75	92.4	94.9	93.6	**0.98**	0.98	0.87
**DPB (SVC)**	**N**	86.3	85.9	86.1	0.93	0.94	0.72	88.0	91.8	89.9	0.97	0.97	0.80
	**C**	83.4	83.2	83.3	0.91	0.93	0.67	88.3	88.3	88.3	0.95	0.96	0.77
	**NC**	86.9	86.4	86.7	0.94	0.95	0.73	93.1	93.8	93.5	**0.98**	0.98	0.87
**AIB (ET)**	**N**	86.9	86.9	86.9	0.94	0.95	0.74	90.0	94.5	92.3	0.97	0.97	0.85
	**C**	84.0	84.8	84.4	0.92	0.93	0.69	88.0	88.0	88.0	0.96	0.96	0.76
	**NC**	86.4	86.6	86.5	0.95	0.95	0.73	92.1	92.4	92.3	**0.98**	0.98	0.85
**PCB (ET)**	**N**	85.8	86.6	86.2	0.94	0.94	0.72	90.7	93.5	92.1	0.97	0.97	0.84
	**C**	83.3	83.9	83.6	0.91	0.92	0.67	89.0	85.2	87.1	0.94	0.95	0.74
	**NC**	86.6	86.5	86.6	0.94	0.95	0.73	94.2	91.4	92.8	**0.98**	0.98	0.86

# Sn: Sensitivity, **Sp:** Specificity, **Acc:** Accuracy, **MCC:** Matthews Correlation Coefficient, **AUC:** Area Under the Receiver Operating Characteristic curve, **AUPRC:** Area Under the Precision-Recall Curve, AAB**:** Amino acid-based binary profile, **DPB:** Dipeptide-based binary profile, **PCB:** Physico-chemical properties based binary profile, **AIB:** Amino-acid indices based binary profile, **ET:** Extra-tree classifier, **SVC:** Support vector classifier
