# Supplementary material for: AntiBP3: A Method for Predicting Antibacterial Peptides against Gram-Positive/Negative/Variable Bacteria
Source: Antibiotics (Basel). 2024 Feb 8;13(2):168. doi: 10.3390/antibiotics13020168 (PMC10885866; doi:10.3390/antibiotics13020168)
Supplement: Supplementary file 1 [file antibiotics-13-00168-s001.zip › Table S6.pdf]

Table S6: Performance of ML-models on training and validation dataset developed using four types of Binary profile-based features of N, C, NC-terminal of gram-variable ABPs

Feature type	Terminal	Training set						Validation set					
		Sn	Sp	Acc	AUC	AUPRC	MCC	Sn	Sp	Acc	AUC	AUPRC	MCC
AAB (SVC)	N	85.2	85.2	85.2	0.92	0.90	0.70	83.9	89.3	86.6	0.93	0.92	0.73
	C	82.2	82.2	82.2	0.89	0.88	0.64	79.4	83.0	81.2	0.89	0.89	0.63
	NC	86.3	86.2	86.3	0.92	0.91	0.73	83.9	90.1	87.0	**0.94**	0.93	0.74
DPB (RF)	N	45.1	45.3	45.2	0.48	0.53	-0.10	64.8	86.1	75.5	0.86	0.81	0.52
	C	80.0	79.4	79.7	0.88	0.87	0.59	76.9	78.7	77.8	0.86	0.86	0.56
	NC	83.5	83.5	83.5	0.91	0.89	0.67	82.5	47.0	64.8	0.67	0.61	0.32
AIB (SVC)	N	85.4	85.1	85.2	0.92	0.90	0.71	83.9	88.8	86.3	0.93	0.92	0.69
	C	82.0	82.7	82.4	0.89	0.88	0.65	79.2	83.6	81.4	0.89	0.89	0.64
	NC	86.4	86.2	86.3	0.92	0.91	0.73	84.5	90.2	87.3	0.94	0.93	0.73
PCB (SVC)	N	84.7	84.8	84.7	0.91	0.90	0.70	84.1	88.9	86.5	0.93	0.92	0.73
	C	81.3	80.9	81.1	0.88	0.87	0.62	80.5	82.4	81.4	0.88	0.89	0.63
	NC	86.3	86.0	86.1	0.92	0.90	0.72	84.8	89.9	87.3	0.94	0.93	0.75

Sn: Sensitivity, **Sp:** Specificity, **Acc:** Accuracy, **MCC:** Matthews Correlation Coefficient, **AUC:** Area Under the Receiver Operating Characteristic curve, **AUPRC:** Area Under the Precision-Recall Curve, AAB**:** Amino acid-based binary profile, **DPB:** Dipeptide-based binary profile, **PCB:** Physico-chemical properties based binary profile, **AIB:** Amino-acid indices based binary profile, **RF:** Random forest classifier, **SVC:** Support vector classifier
