# Supplementary material for: AntiBP3: A Method for Predicting Antibacterial Peptides against Gram-Positive/Negative/Variable Bacteria
Source: Antibiotics (Basel). 2024 Feb 8;13(2):168. doi: 10.3390/antibiotics13020168 (PMC10885866; doi:10.3390/antibiotics13020168)
Supplement: Supplementary file 1 [file antibiotics-13-00168-s001.zip › Table S7.pdf]

Table S7: Performance of ML-models on training and validation dataset developed using FastText based features for GP, GN and GV ABPs

Category of ABPs	Feature (n-gram size)	Training set						Validation set					
		Sn	Sp	Acc	AUC	AUPRC	MCC	Sn	Sp	Acc	AUC	AUPRC	MCC
Gram-positive	1g(ET)	83.2	83.9	83.5	0.91	0.91	0.67	80.7	87.6	84.1	0.92	0.91	0.68
	2g(ET)	82.4	83.1	82.7	0.91	0.92	0.66	81.2	89.8	85.5	0.91	0.91	0.71
	3g(LR)	78.8	78.0	78.4	0.87	0.88	0.57	72.6	83.3	78.0	0.84	0.85	0.56
	2g & 3g combined (ET)	83.1	82.7	82.9	0.91	0.92	0.66	79.6	85.5	82.5	0.90	0.90	0.65
Gram-negative	1g(ET)	88.5	88.8	88.6	0.95	0.96	0.77	92.4	91.1	91.8	0.97	0.98	0.84
	2g(ET)	87.3	87.8	87.5	0.94	0.95	0.75	92.1	90.0	91.1	0.97	0.98	0.82
	3g(LR)	84.6	85.1	84.9	0.92	0.93	0.70	91.4	90.0	90.7	0.96	0.96	0.82
	2g & 3g combined (ET)	88.4	88.7	88.5	0.95	0.96	0.77	91.8	89.7	90.7	0.97	0.98	0.82
Gram-variable	1g(SVC)	81.8	82.2	82.0	0.89	0.86	0.64	84.5	86.6	85.5	0.92	0.92	0.71
	2g(SVC)	85.7	85.4	85.5	0.92	0.90	0.71	84.5	89.3	86.9	0.93	0.93	0.74
	3g(LR)	84.0	83.9	84.0	0.91	0.89	0.68	81.9	86.1	84.0	0.92	0.91	0.68
	2g &3g combined (SVC)	86.6	86.2	86.4	0.92	0.91	0.73	83.9	89.2	86.6	0.93	0.92	0.73

Sn: Sensitivity, **Sp:** Specificity, **Acc:** Accuracy, **MCC:** Matthews Correlation Coefficient, **AUC:** Area Under the Receiver Operating Characteristic curve, **AUPRC:** Area Under the Precision-Recall Curve
