# Supplementary material for: AntiBP3: A Method for Predicting Antibacterial Peptides against Gram-Positive/Negative/Variable Bacteria
Source: Antibiotics (Basel). 2024 Feb 8;13(2):168. doi: 10.3390/antibiotics13020168 (PMC10885866; doi:10.3390/antibiotics13020168)
Supplement: Supplementary file 1 [file antibiotics-13-00168-s001.zip › Table S8.pdf]

Table S8: Performance of hybrid model (BLAST+ML) on the validation set of gram-positive ABPs independent at different e-value

E-value	Correct hits (BLAST+ML)	Sn	Sp	Acc	AUC	AUPRC	MCC
**1.00E-20**	**86.02%**	**81.7**	**90.3**	**86.0**	**0.94**	**0.93**	**0.72**
**1.00E-10**	85.75%	81.2	90.3	85.8	0.93	0.92	0.72
**1.00E-06**	86.29%	82.3	90.3	86.3	0.93	0.91	0.73
**0.0001**	86.83%	83.3	90.3	86.8	0.93	0.91	0.74
**0.001**	87.90%	85.5	90.3	87.9	0.93	0.91	0.76
**0.01**	88.44%	86.0	90.9	88.4	0.93	0.92	0.77
**0.1**	87.90%	86.6	89.3	87.9	0.93	0.92	0.76
**1**	86.56%	87.1	86.0	86.6	0.93	0.92	0.73
**10**	81.18%	83.9	78.5	81.2	0.90	0.89	0.62
**50**	80.11%	83.3	76.9	80.1	0.90	0.89	0.60
**100**	80.38%	83.3	77.4	80.4	0.90	0.89	0.61
**200**	80.11%	83.3	76.9	80.1	0.90	0.89	0.60
**1000**	80.38%	83.9	76.9	80.4	0.91	0.89	0.61
**Only ML**	85.48%	80.7	90.3	85.5	0.93	0.92	0.71

Sn: Sensitivity, **Sp:** Specificity, **Acc:** Accuracy, **MCC:** Matthews Correlation Coefficient, **AUC:** Area Under the Receiver Operating Characteristic curve, **AUPRC:** Area Under the Precision-Recall Curve
