# Supplementary material for: AntiBP3: A Method for Predicting Antibacterial Peptides against Gram-Positive/Negative/Variable Bacteria
Source: Antibiotics (Basel). 2024 Feb 8;13(2):168. doi: 10.3390/antibiotics13020168 (PMC10885866; doi:10.3390/antibiotics13020168)
Supplement: Supplementary file 1 [file antibiotics-13-00168-s001.zip › Table S9.pdf]

Table S9: Performance of hybrid model (BLAST+ML) on the validation set of gram-negative ABPs independent at different e-value

E-value	Correct hits (BLAST+ML)	Sn	Sp	Acc	AUC	AUPRC	MCC
**1.00E-20**	93.30%	92.4	94.2	93.3	0.98	0.98	0.87
**1.00E-10**	93.99%	94.2	93.8	94.0	0.98	0.97	0.88
**1.00E-06**	94.16%	94.5	93.8	94.2	0.97	0.97	0.88
**0.0001**	94.16%	94.2	94.2	94.2	0.97	0.97	0.88
**0.001**	94.33%	94.5	94.2	94.3	0.97	0.97	0.89
**0.01**	94.67%	94.9	94.5	94.7	0.97	0.97	0.89
**0.1**	94.85%	94.9	94.9	94.9	0.97	0.97	0.90
**1**	94.16%	93.8	94.5	94.2	0.97	0.97	0.88
**10**	91.41%	92.1	90.7	91.4	0.96	0.96	0.83
**50**	90.21%	90.7	89.7	90.2	0.96	0.96	0.80
**100**	90.03%	90.4	89.7	90.0	0.96	0.96	0.80
**200**	89.86%	90.0	89.7	89.9	0.96	0.96	0.80
**1000**	89.18%	88.7	89.7	89.2	0.96	0.96	0.78
**Only ML**	93.64%	92.4	94.2	93.3	0.98	0.98	0.87

Sn: Sensitivity, **Sp:** Specificity, **Acc:** Accuracy, **MCC:** Matthews Correlation Coefficient, **AUC:** Area Under the Receiver Operating Characteristic curve, **AUPRC:** Area Under the Precision-Recall Curve
